# Central cholinergic signal-mediated neuroendocrine regulation of vasopressin and oxytocin in ovine fetuses

**DOI:** 10.1186/1471-213X-8-95

**Published:** 2008-10-02

**Authors:** Lijun Shi, Caiping Mao, Fanxing Zeng, Yuying Zhang, Zhice Xu

**Affiliations:** 1 Department of Human Sport Science, Beijing Sport University, Beijing 100084, PR China; 2 Perinatal Biology Center, Soochow University School of Medicine, Suzhou 215007, PR China; 3 Center for Perinatal Biology, Loma Linda University School of Medicine, Loma Linda, California 92350, USA

## Abstract

**Background:**

The hypothalamic-neurohypophysial system plays a fundamental role in the maintenance of body fluid homeostasis by secreting arginine vasopressin (AVP) and oxytocin (OT) in response to a variety of signals, including osmotic and nonosmotic stimuli. It is well established that central cholinergic mechanisms are critical in the regulation of cardiovascular responses and maintenance of body fluid homeostasis in adults. Our recent study demonstrated that intracerebroventricular (i.c.v.) injection of carbachol elicited an increase of blood pressure in the near-term ovine fetuses. However, *in utero *development of brain cholinergic mechanisms in the regulation of the hypothalamic neuropeptides is largely unknown. This study investigated AVP and OT neural activation in the fetal hypothalamus induced by central carbachol.

**Results:**

Chronically prepared near-term ovine fetuses (0.9 gestation) received an i.c.v. carbachol (3 μg/kg). Fetal blood samples were collected for AVP and OT assay, and brains were used for c-fos mapping studies. I.c.v. carbachol significantly increased fetal plasma AVP and OT concentrations. Intense FOS immunoreactivity (FOS-ir) was observed in the fetal supraoptic nuclei (SON) and paraventricular nuclei (PVN) in the hypothalamus. Double labeling demonstrated that a number of AVP- and OT-containing neurons in the fetal SON and PVN were expressing c-fos in response to central carbachol.

**Conclusion:**

The results indicate that the central cholinergic mechanism is established and functional in the regulation of the hypothalamic neuropeptides during the final trimester of pregnancy. This provides evidence for a functional link between the development of central cholinergic mechanisms and hypothalamic neuropeptide systems in the fetus.

## Background

The central nervous system (CNS) contains extensive cholinergic components [1], and central cholinergic pathways play an important role in the control of cardiovascular and body fluid homeostasis [2-6]. Carbachol, as a cholinergic agonist elicits a variety of neurobiological and physiological responses, including pressor responses, thirst, natriuresis as well as secretion and release of arginine vasopressin (AVP) in adult models [1,7-11].

AVP and oxytocin (OT) are two major neuropeptides synthesized and released by magnocellular neurosecretory neurons in the hypothalamus. Both of them are involved in vascular functions and hydromineral homeostasis [12]. A variety of signals, including the central cholinergic stimulation, can cause release of AVP and OT in adults [13,14]. It is unknown however whether central cholinergic mechanisms have been developed *in utero *in the control of AVP and OT systems in the fetal hypothalamus. Addressing this question is important to both prenatal and postnatal health problems. Despite this, our recent study has shown that intracerebroventricular (i.c.v.) injection of carbachol produces reliable pressor responses [15] accompanied by a bradycardia in ovine fetuses at near-term. Both nicotine and muscarinic receptors have been detected in the fetal brain [16-20]. These results indicate that the central cholinergic mechanism-mediated cardiovascular regulation is functional *in utero*. In addition, studies from other lines have shown that various signals, including angiotensin II, osmotic and hypovolemic stimulation, can induce AVP secretion and release from the hypothalamus and pituitary [21-26]. The evidence from these two lines of research has independently proved that central cholinergic systems have developed in the last third of gestation, and the AVP system as a model of hypothalamic neuropeptide pathways has matured enough in prenatal neuroendocrine regulation. However, a link or a functional bridge between those two parts of studies has not been established in fetuses. Thus, the present study was designed to fill the gap and build an initial link between these two lines of studies.

Based on available information, it is reasonable to hypothesize that fetal brain cholinergic mechanisms-mediated hypothalamic neuropeptide regulation is established before birth. To test this hypothesis we used a chronically prepared ovine fetal model. Under the unstressful and unanaesthetised condition after surgical recovery, ovine fetuses were tested following i.c.v. carbachol *in utero*. We determined the neural activity in the hypothalamic nuclei, the fetal supraoptic nuclei (SON) and paraventricular nuclei (PVN), under the condition of presence of the central cholinergic signal. We also collected fetal blood samples at real time during i.c.v. injection periods for measuring plasma AVP and OT levels. Additionally, we characterized hypothalamic activated cells induced by the central cholinergic signal. Information gained is helpful to establish a functional "bridge" between the functional development of the brain cholinergic mechanism and the hypothalamic neuropeptide systems.

## Results

### Blood values

Histological analysis confirmed that all i.c.v. cannulae were inserted into the fetal lateral vehicle. For both the control and experimental animals, i.c.v. injection of carbachol (3 μg/kg) or vehicle had no effect on maternal or fetal plasma osmolality, Na^+^, K^+^, and Cl^- ^concentrations, or arterial blood pH, PO_2_, PCO_2_, hemoglobin, and hematocrit (all *P *> 0.05). All arterial values were within normal ranges and were not different between the control and experimental groups (all *P *> 0.05, Table 1).

**Table 1:** Fetal and maternal arterial values before and after intracerebroventricular carbachol injection into the fetal brain.

	**baseline**	**15 min**	**30 min**	**60 min**	**90 min**
**Fetus**					
pH	7.37 ± 0.01	7.36 ± 0.01	7.35 ± 0.01	7.35 ± 0.01	7.36 ± 0.01
PCO_2 _(mmHg)	47.6 ± 2.0	47.4 ± 1.8	48.3 ± 1.7	48.0 ± 1.6	48.6 ± 1.4
PO_2 _(mmHg)	21.2 ± 0.6	20.4 ± 1.5	20.6 ± 1.3	20.4 ± 1.4	20.5 ± 1.2
Hct (%)	25.1 ± 0.5	26.1 ± 1.0	26.5 ± 1.4	27.2 ± 1.5	26.9 ± 1.2
Hb (g/dL)	8.2 ± 0.4	8.6 ± 0.4	8.6 ± 0.6	8.5 ± 0.5	8.3 ± 0.4
Osmolality(mOsmol/kg)	302.6 ± 1.3	304.2 ± 1.7	303.8 ± 1.6	302.4 ± 1.6	303.4 ± 1.4
Na^+ ^(mEq/L)	142.8 ± 0.9	142.5 ± 0.6	142.8 ± 0.7	142.6 ± 0.8	143.1 ± 0.6
K^+ ^(mEq/L)	4.7 ± 0.2	4.6 ± 0.2	4.6 ± 0.1	4.6 ± 0.2	4.5 ± 0.2
Cl^- ^(mEq/L)	109.7 ± 0.6	109.7 ± 0.7	109.3 ± 0.9	109.3 ± 0.8	109.4 ± 0.6
					
**Maternal**					
pH	7.44 ± 0.05	7.44 ± 0.06	7.43 ± 0.05	7.44 ± 0.04	7.43 ± 0.07
PCO_2 _(mmHg)	33.7 ± 0.5	34.0 ± 3.3	34.5 ± 2.6	32.6 ± 0.9	34.8 ± 1.2
PO_2 _(mmHg)	113.4 ± 3.6	114.3 ± 2.6	114.0 ± 4.6	115.5 ± 3.2	117.5 ± 4.2
Hct (%)	28.3 ± 0.6	27.8 ± 1.0	27.1 ± 0.6	27.9 ± 0.9	27.6 ± 1.0
Hb (g/dL)	7.9 ± 0.4	8.1 ± 0.4	8.1 ± 0.6	7.7 ± 0.5	7.9 ± 0.4
Osmolality(mOsmol/kg)	304.2 ± 0.6	305.2 ± 1.2	306.6 ± 1.4	306.2 ± 1.5	305.6 ± 1.5
Na^+ ^(mEq/L)	148.0 ± 0.8	147.9 ± 0.8	147.8 ± 06	147.6 ± 0.6	147.6 ± 0.6
K^+ ^(mEq/L)	4.0 ± 0.1	4.0 ± 0.1	4.0 ± 0.1	4.1 ± 0.1	4.1 ± 0.1
Cl^- ^(mEq/L)	113.4 ± 0.8	113.2 ± 0.7	113.1 ± 0.7	113.2 ± 0.9	113.5 ± 1.1

Upper panel, fetal arterial values; lower panel, maternal arterial values. Carbachol, 3 μg/kg. Values are mean ± SEM. Hct: hematocrit. Hb: hemoglobin.

Consistent with our previous studies [15], there was no difference in maternal mean arterial pressure between control and experimental groups (F_8,1 _= 0.12, *P *> 0.05). However, i.c.v. injection of carbachol significantly increased fetal mean arterial pressure (F_8,1 _= 15.42, *P *< 0.01). Fetal mean arterial pressure (F_8,1 _= 27.80, *P *< 0.01) was increased and heart rate (F_8,1 _= 12.51, *P *< 0.01) was decreased following central administration of carbachol (data not shown).

### Plasma AVP and OT assay

**Figure 1:**
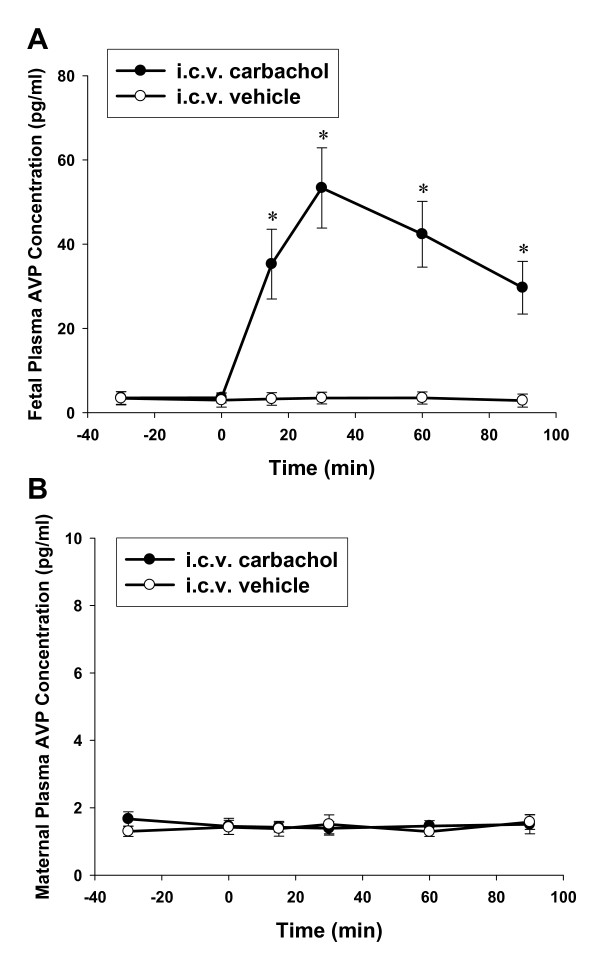
**The effect of i.c.v. carbachol on fetal (A) and maternal (B) plasma AVP concentration**. The dose of carbachol, 3 μg/kg; 0 min: time for i.c.v. injection. *, *P *< 0.01 compared with the baseline level. AVP: arginine vasopressin.

**Figure 2:**
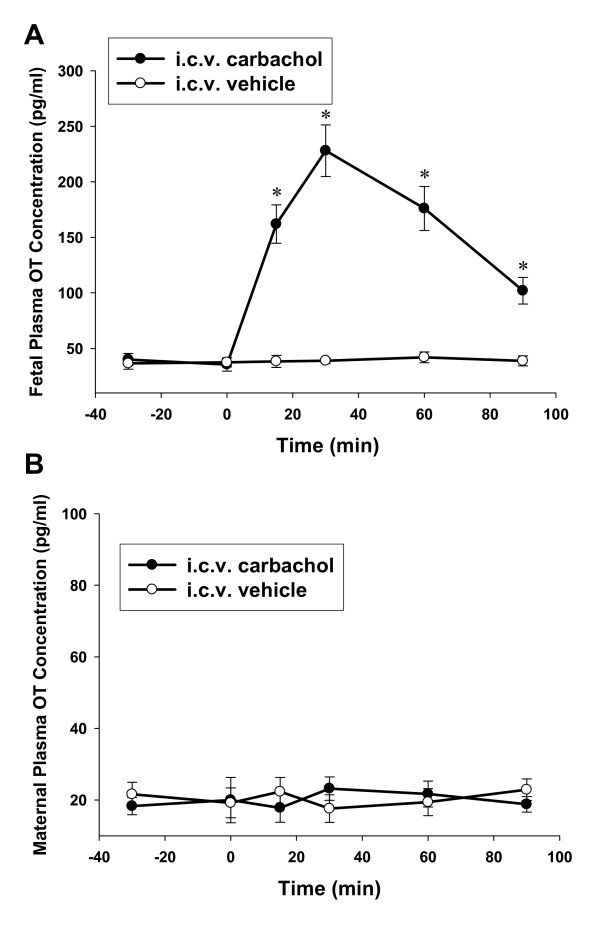
**The effect of i.c.v. carbachol on fetal (A) and maternal (B) plasma OT concentration**. The dose of carbachol, 3 μg/kg; 0 min: time for i.c.v. injection. *, *P *< 0.01 compared with the baseline level. OT: oxytocin.

There was no significant difference in plasma AVP and OT levels between the control and the experimental ewes to i.c.v. injection (AVP: F_8,1 _= 0.37, OT: F_8,1 _= 0.23, both *P *> 0.05). However, the fetal AVP and OT concentrations were significantly increased after i.c.v. carbachol administration (AVP: F_8,1 _= 73.60, OT: F_8,1 _= 58.61, *P *< 0.01). In the control group, i.c.v. injection of the vehicle did not change the maternal and fetal plasma AVP or OT levels. The i.c.v. carbachol significantly increased fetal plasma AVP and OT levels (AVP: F_28,6 _= 43.23; OT: F_28,6 _= 35.40, respectively, both *P *< 0.01, the baseline period vs the period after i.c.v. injection) (Figure 1 and 2). Fetal plasma AVP and OT increased significantly within 15 minutes after i.c.v. carbachol, and the peak levels of plasma AVP and OT were observed at 30 minutes (AVP: from base line level 3.5 ± 1.2 to 53.4 ± 9.5 pg/ml; OT: from base line level 35.6 ± 6.0 to 228.3 ± 23.2 pg/ml) after injection of carbachol. There was no change in maternal plasma AVP and OT after i.c.v. injection of carbachol into the fetus.

### FOS-immunoreactivity and double labeling

**Figure 3:**
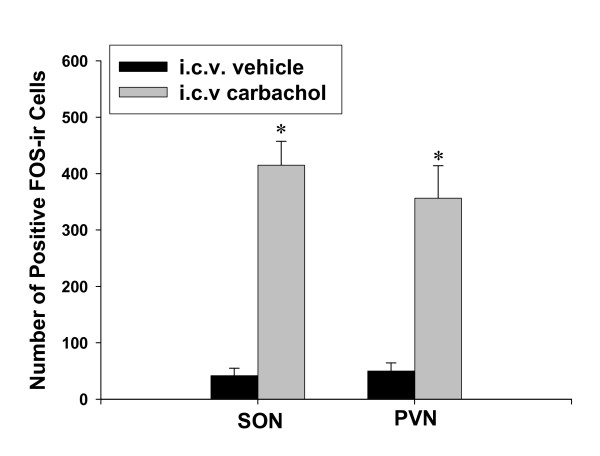
**The effect i.c.v. injection of carbachol on FOS-ir in the fetal hypothalamus**. The dose of carbachol, 3 μg/kg; SON: the supraoptic nuclei; PVN: the paraventricular nuclei. *, *P *< 0.01 compared with the control level.

**Figure 4:**
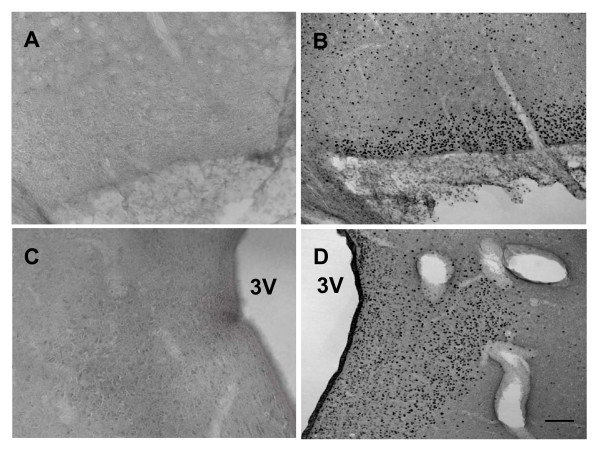
**FOS-ir induced by i.c.v. carbachol in the fetal SON and PVN**. SON (upper panel) and PVN (bottom panel). A and C: the animal treated with i.c.v. vehicle; B and D: the animal injected with i.c.v. carbachol (3 μg/kg). 3V: the third ventricle. Scale bar = 200 μm.

In the control fetuses, there was no or few FOS-ir in the basal forebrain structures. However, i.c.v. carbachol produced intense FOS-ir in the fetal forebrain, including the SON and PVN in the hypothalamus (Figure 3 and 4). There was a significant difference of FOS-ir in the SON and PVN between the i.c.v. vehicle and the i.c.v. carbachol injected fetuses (t = 11.2 and 8.9, respectively, both *P *< 0.01).

**Figure 5:**
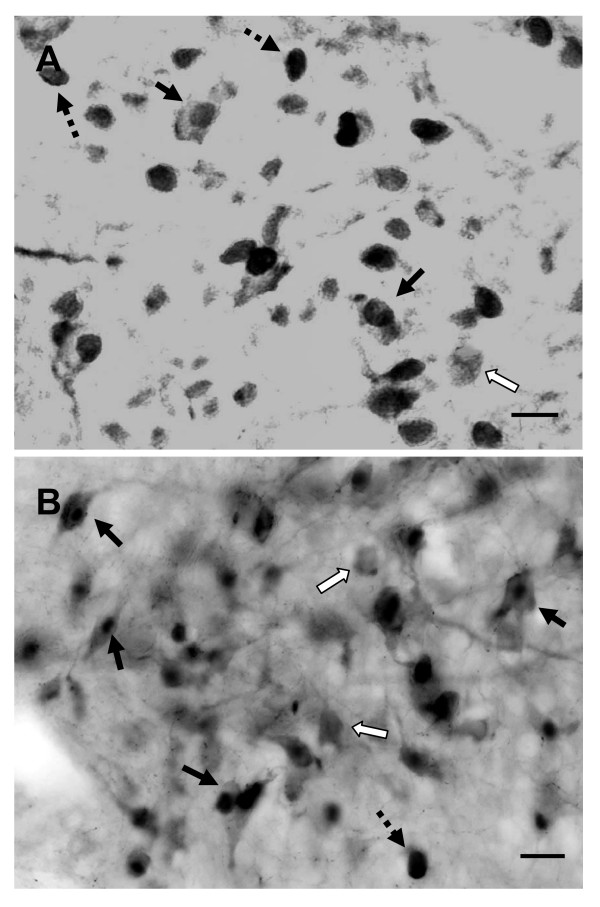
**FOS-ir and AVP-ir (or OT-ir) in the SON and PVN following i.c.v. carbachol**. A: double labeling of FOS-ir and AVP-ir in the SON. B: double labeling of FOS-ir and OT-ir in the PVN. Solid arrows indicate co-localization of FOS-ir and AVP-ir (or OT-ir). Open arrows indicate AVP-ir (or OT-ir) positive cells without FOS-ir. Dash arrows indicate positive FOS-ir outside of AVP- (or OT-ir) containing cells. Scale bar = 10 μm.

Double labeling showed that there was no FOS-ir in AVP-containing and OT-containing cells in the brain of control fetuses. However, co-localization of FOS-ir in many AVP and OT neurons was detected in both sides of the SON and PVN following i.c.v. carbachol in the fetal hypothalamus (Figure 5). Although many AVP and OT neurons were co-localized with positive FOS-ir, there were some AVP and OT cells not labeled with FOS-ir. There was no difference in the total counting of AVP-ir and OT-ir in the SON and PVN between the control and the treated fetuses (all *P *> 0.05). Double labeling of FOS-ir and AVP-ir in the SON was significantly higher in the carbachol-treated fetuses than in the control animals (*t *= 6.78, *P *< 0.01). 82% of the AVP (+) neurons in the SON were FOS (+) in the carbachol treated fetuses. However, very few of FOS (+) cells in the SON (about 6%) were not AVP-containing neurons. Also, double labeling of FOS-ir and OT-ir in the PVN was significantly higher in the carbachol-treated fetuses than in the control animals (*t *= 5.66, *P *< 0.01). 54% of the OT (+) neurons in the PVN were FOS (+) in the carbachol treated fetuses, and only 8% of FOS (+) cells in the PVN were not OT-containing neurons.

## Discussion

The present study provides new insight that the central cholinergic agonist causes AVP and OT release in the ovine fetuses. The finding that c-fos expression was induced in fetal AVP and OT neurons is the evidence that the hypothalamic pathway is activated and cholinergic mechanisms are functional in the control of fetal neuroendocrine function at 0.9 gestation.

Although there are evidence regarding to neuroendocrinological responses induced by central cholinergic stimulation in adults, to our knowledge, whether central cholinergic mechanisms have developed *in utero *on the control of the neuropeptide release from the fetal hypothalamic-hypophysial system has been to date unknown. To address this issue, two essential questions must be considered for the establishment of functional relationship between cholinergic systems and hypothalamic neuropeptide systems in the fetal brain. Firstly, cholinergic binding sites must be intact and functional; and secondly, the hypothalamic neuropeptide network must mature enough in response to cholinergic signals. To answer the first question, previous studies have demonstrated both nicotinic and muscarinic receptors have developed in the fetal brain [16-20]; our recent study has shown that i.c.v. injection of carbachol produces reliable pressor responses [15] in ovine fetuses at near-term, indicating that the central cholinergic mechanisms-mediated cardiovascular regulation is functional *in utero*. To answer the second question, by using neuroanatomical, immunochemical, and molecular techniques, a variety of hypothalamic neuropeptides such as corticotropin releasing hormone (CRH), gonadotropin releasing hormone, growth hormone releasing hormone, luteinizing hormone releasing hormone, somatostatin, neurophysin, neuropeptide Y were detected in the fetal hypothalamus [27-29]. For example, hypothalamic CRH expression was increased at the end of gestation [30], and the hypothalamic-pituitary-adrenal (HPA) axis exists at late gestational period [30,31]. Thus, the evidence from those two lines of research has independently proven that central cholinergic receptor systems have well developed at least at late gestation, and many neuropeptides pathways are matured enough in prenatal neuroendocrine regulation. However, a link or a functional bridge between those two parts of studies has so far not been established. In light of this, the new findings from the present study may fill this gap.

AVP and OT are two main neuropeptides synthesized by magnocellular neurosecretory neurons in the SON and PVN in the hypothalamus. Previous studies in fetal models have shown that the hypothalamic AVP and OT systems are relatively intact before birth [21-23,32], and various signals, including angiotensin II, osmotic and hypovolemic stimulation, can induce AVP secretion from the fetal hypothalamus [21-26]. Thus, it should not be strange to use the AVP and OT system as a model to study hypothalamic neuropeptides in fetuses.

In addition to the main physiological effect of antidiuresis, the peripheral AVP also participate in the vasoconstriction and pressor responses in both adults and fetuses [24,33]. In the present study, we demonstrated that i.c.v. injection of cholinergic agonist carbachol induced a marked increase of fetal plasma AVP concentrations without influence on maternal AVP levels. The fetal AVP in circulation reached the peak in 30 mins which is about 15 fold of the baseline level. Associated with the plasma AVP elevation, the fetal arterial blood pressure also increases significantly. Whether increased AVP levels contributed to pressor responses still needs further studies.

In comparison to studies on fetal AVP, the present study was the first experiment using central injection approach *in utero *to investigate the hypothalamic OT system. OT is an important hormone not only inducing sexual and maternal behavior, but also be involved in vascular and cardiac relaxation and hydromineral homeostasis [34-36]. Previous studies indicated that fetal plasma OT concentrations changed in sheep with the gestation age. At the last third of gestation, the fetal plasma OT levels were usually higher than maternal OT levels [37]. Our results showed that the baseline level of fetal plasma OT level is 35.6 ± 6.0 pg/ml, which is significantly higher than the maternal levels (20.0 ± 5.8 pg/ml) at near-term gestation. After i.c.v. injection of carbachol, fetal plasma OT increased about six times of the baseline level and reached the maximum within 30 mins. The increase of OT lasted for more than 90 mins. Similar to AVP, the lack of change of the maternal plasma OT suggests that the increased fetal plasma OT is released from its own nerurohypophysis in response to central carbachol and fetal OT seems not pass the placental barrier to the maternal side. Notably, the increased fetal plasma OT by i.c.v. carbachol is less than the increased of AVP compared to the baseline, which is quite different from adults in the same neuropeptide responses [13]. Lauand *et al *reported that under the same condition of carbachol stimulation, the fold of increased AVP and OT was similar [13]. While in our fetuses, it seems the AVP neurons had a lower response threshold than OT neurons to cholinergic stimulation.

Previous studies on the initiation of parturition show that fetal OT may play an important role in signal processing of labor. Fetal OT may initiate or accelerate the course of labor, while fetal AVP plays a role in the adaptation to stress-caused by the birth process-by redistribution of the fetal blood flow [38]. OT has been suggested to be involved in the regulation of adrenocorticotropic hormone (ACTH) secretion in fetal sheep in late gestation [32], and the latter plays an important role in the onset of spontaneous birth. With permanent bilateral PVN lesions placed at 106–110 or 118–122 days of gestation, the most apparent physiological change is that the fetal sheep fails to deliver [39,40]. These observations demonstrate that the fetal brain structure, PVN, is necessary for parturition to occur. The PVN is not only abundant of CRH neurons but also the main source for OT synthesis. Therefore, a sharp increase of OT in the fetal body could be viewed as a critical signal for giving birth, and this hormone contributes partially to initiation of labor. We speculate that certain protective mechanisms may exist in premature fetuses to prevent dramatically increasing of OT to initiate premature labor. This could be one of reasons why the released OT is relatively less than AVP under the same condition in the fetus compared to that in adults, indicting that fetal OT neurons showed a lower responsive ability in secretion or release of OT into the circulation from the fetal brain. However, additional studies are needed for to clarify this further.

In light of this, one important significance of the increased OT by the central cholinergic signal in the present study is linked to clinical problem-premature birth. If fetal central cholinergic mechanism is over activated by any factor at the wrong time (before term), it is very likely to induce premature labor due to a significant increase of fetal plasma OT levels. It is clear that many environmental factors or maternal conditions such as smoking may increase cholinergic activity in the fetal CNS. Therefore, those environmental and maternal factors could also subsequently produce a higher level of OT *in utero *according to the new finding in the present study. Therefore, to prevent of premature birth, one may need to pay attention to factors that can increase central cholinergic activity in the fetal brain.

Previous studies in adult animals showed that hyperosmolality is a potent stimulus in the control of AVP and OT secretion [41-43]. A significant correlation has been found between plasma osmolality and AVP and OT concentrations following intraperitoneal or intravenous administration of hypertonic NaCl [25,44]. However, in the present study, the fetal physiological status remained stable after surgical recovery and under the condition of i.c.v. injection of carbachol as arterial values (pH, PCO_2_, PO_2_, and plasma electrolytes) were not changed. Consequently, unchanged fetal plasma osmolality and sodium levels make it unlikely that the central carbachol induced release of fetal AVP and OT was due to osmotic mechanisms.

In addition to hyperosmolar stimulus, hypovolemia and hypotension also can enhance the release of AVP and OT [35,45]. However, in the present study, fetal blood hematocrit and hemoglobin were not affected by the central carbachol, indicating unchanged fluid volume. Furthermore, if there was decrease of extracellular volume, the arterial blood pressure would be subsequently lowered. However, the results of pronounced pressor responses in the present as well as previous study [15] after the i.c.v. carbachol do not support the possibility that volume mechanisms may contribute to AVP and OT release by central cholinergic stimulation *in utero*.

Hypoxemia is also reported as being a potent stimulus of fetal AVP secretion [46,47]. The fetal arterial PO_2 _is stable before and after i.c.v. carbachol, therefore can excluding this possibility.

A more likely explanation for the elevation of the fetal plasma AVP and OT is the facilitation by central cholinergic stimuli *per se*. Central carbachol activates neural pathways in the brain. The significant enhancement of activity of fetal AVP and OT neurons suggests that central cholinergic mechanisms is functional in the control of the hypothalamic neuropeptide systems. Combining the results of plasma AVP and OT changes following i.c.v. carbachol, we can get at least three preliminary conclusions: (1) the fetal hypothalamic OT system is functional intact at least at near-term; (2) central cholinergic signals play a role in the control of release of both AVP and OT from the fetal brain; and (3) the network or bridge between central cholinergic signals and the hypothalamic neuropeptide systems have been developed at 0.9 gestation *in utero*.

Several lines of evidence showed that multiple sites in the CNS play a role in cholinergically mediated neuroendocrine regulation, and that regulation by centrally administered cholinergic agonists are site-specific. To determine central actions of carbachol in the fetal hypothalamus, a c-fos mapping was employed in this study. FOS protein immunoreactivity has been widely used as a marker of neuronal activation [48,49] following i.c.v. injection of neuroactive substances including carbachol [1,13]. In the present study, a number of neurons or cells were activated with the expression of c-fos in both PVN and SON in the fetal hypothalamus following i.c.v. carbachol. A question was then raised: what kind of neurons or neurosecretary cells in the fetal hypothalamus responded to the cholinergic signal? In addressing this question, we used double labeling experiments to characterize the hypothalamic activated cells. Notably, three phenomena were observed: (1) co-localization of FOS-ir and AVP-ir or OT-ir; (2) AVP-ir or OT-ir positive while FOS-ir negative; (3) FOS-ir positive while AVP-ir and OT-ir negative. The first phenomenon demonstrates that many AVP- or OT-containing neurons were activated. Regardless of whether the AVP and OT neurons in the fetal hypothalamus were activated by carbachol directly or via other pathways indirectly following the i.c.v. carbachol, the c-fos expression induced in these nuclei is evidence that central AVP and OT systems are relatively intact and sufficiently functional to cholinergic stimulation at near-term. The elevation of plasma AVP and OT concentrations is further support for the central neuron activation. The second phenomenon indicates that some of the AVP and OT neurons do not express FOS-ir following carbachol stimulation. Although numerous evidence from previous studies support that FOS acts as a marker of neuron activation, caution should be taken when negative expression of c-fos appears in experiments. There could be two possibilities, one was that these magnocellular neurons were not activated; the other was they might be activated but did not express FOS protein. The third phenomenon suggests that c-fos located in other activated cells that were neurons other than vasopressinergic and oxytocinergic cells.

Although we mainly focused on the fetal hypothalamus in this study, our previous study [15] showed that the similar i.c.v. cholinergic stimulation also can induce cellular activation in the fetal anterior third ventricle (AV3V) region (including the median preoptic nucleus and organum vasculosum of the lamina terminalis) in the forebrain, and the areas (such as the area postrema, lateral parabrachial nucleus, nucleus tractus solitary, and rostral ventrolateral medulla) in the hindbrain of the ovine fetus. Those central structures have rich connections with the hypothalamic PVN and SON in adults. Further studies are required to elucidate the activation of AVP and OT neurons in the present study was due to direct, or indirect, or both routes by central carbachol. The results gained will provide additional information on the development of central pathways related to cholinergic signals.

## Conclusion

The present study demonstrated, for the first time, that i.c.v. carbachol could induce fetal AVP and OT release at 0.9 gestation, indicating functional maturation of the central cholinergic mechanism-mediated neuroendocrine secretion in the fetal hypothalamus. The hormonal responses are associated with activation of AVP and OT neurons in the fetal SON and PVN, confirming that the hypothalamic-neurohypophysial system has been well developed and plays important roles in the cholinergic-mediated neuroendocrine regulation before birth. This novel and important data increases our understanding of the functional development of brain cholinergic mechanisms in the control of neuropeptide *in utero*. The present data not only provide evidence of the functional developmental picture of brain hypothalamic-neurohypophysial systems in response to cholinergic signals at 0.9 gestation, but also offers a useful experimental model in study of the development of hypothalamic pathways in respond to central neuroactive substances *in utero*.

## Methods

### Animals and surgical preparation

Time-dated pregnant ewes with singleton fetuses (gestational age 130 ± 3, term ~145 days) were used. The ovine were housed indoors in individual steel study cages and acclimated to a 12:12-h light-dark cycle. Both food and water were provided ad libitum, except for the last 24 h before surgery, when food was withheld. All protocols in this study were approved by Soochow University School of Medicine Animal Care Committee.

Surgery was performed under strict aseptic conditions. Anesthesia was induced by an intramuscular injection of ketamine hydrochloride (20 mg/kg) plus atropine sulfate (50 μg/kg) and was maintained by maternal endotracheal ventilation with 1 L/min oxygen and 3% isoflurane. Polyethylene catheters (ID = 1.8 mm, OD = 2.3 mm) were inserted into a maternal femoral vein and artery and advanced into the inferior cava and abdominal aorta, respectively. The uterus was exposed by a midline abdominal incision, and a small hysterotomy was performed to provide access to fetal hindlimbs. Polyethylene catheters (ID = 1.0 mm, OD = 1.8 mm) were inserted into the femoral vein and artery. After closure of this initial uterine incision, a second uterine incision was made over the fetal head. A midline incision was made to expose the skull and an intracranial cannula (18 gauge) was placed in the fetal lateral ventricle and held in place with dental cement. The coordinates for cannula placement were: anterior-posterior: +0.1 cm in t front of the bregma; medial-lateral: 0.8 cm from the middle line; and ventral: 1.8 cm below the dura. Patency of the catheter at insertion was assessed by free flow of cerebrospinal fluid via gravity drainage (the placement of cannula was ultimately verified by histological analysis following kill of each animal). An intrauterine catheter was inserted for measuring amniotic fluid pressure. The uterine incisions were closed in layers. All catheters were passed through a subcutaneous tunnel and exteriorized through a small incision on the ewe's flank. Catheters were kept in a cloth pouch attached to the ewe's flank and filled with heparinized saline.

Five days of postoperative recovery were allowed before experimental studies. Antibiotics were administered intravenously twice daily to the ewe (70 mg gentamicin and 1 g oxacillin) and to the fetus (8 mg gentamicin and 30 mg oxacillin) during the first 2–3 days postsurgery.

### Experimental protocol

All experiments were performed on conscious animals standing in their holding cages, with food and water provided ad libitum. Studies began with a baseline period (-60 to 0 minutes) followed by experimental period (0 to 120 minutes) in two groups (control: n = 5; experimental: n = 5). Beginning at time 0, carbachol (3 μg/kg, Sigma) in isotonic saline (1 ml) was injected i.c.v. into the fetus over 5 minutes. Drug doses were based on estimated fetal body weight [50]. To the control animals, isotonic saline (vehicle) was injected. Throughout the basal and experimental periods, maternal and fetal arterial blood was withdrawn at timed intervals for measurement of pH, blood gases, hematocrit, plasma electrolyte composition, osmolality, AVP and OT concentrations. Fetal blood samples were replaced with an equivalent volume of heparinized maternal blood withdrawn before the study. Blood PO_2_, PCO_2_, and pH were measured at 39°C with a Nova analyzer system (AD Instruments, Australia). Plasma and urinary osmolality was measured by freezing-point depression on an Advanced Digimatic osmometer (Advanced Instruments, Needham Heights, MA).

Throughout the study, maternal and fetal systolic and diastolic pressure, amniotic fluid pressure, and heart rate were monitored. The fetal mean arterial pressure (MAP) was corrected for amniotic cavity pressure.

### Hormone Experiments

The maternal and fetal blood samples were collected into iced tubes containing lithium heparin during the baseline and the experimental periods. Blood samples for AVP and OT assays were centrifuged immediately. The plasma was then stored at -20°C before radioimmunoassay (RIA). Plasma AVP and OT concentrations were measured using Sep-Pak C18 cartridge extraction. Samples for AVP and OT extraction were acidified with 1N HCl and extracted. Acidified plasma samples were added slowly to the columns, and the columns were washed with 0.1% trifluoroacetic acid. The absorbed AVP and OT were eluted with 50% methanol and 0.1% TFA, and the eluates were dried in a Speed-Vac concentrator. The assay sensitivity was 1.2 pg for AVP/tube and 1.6 pg for OT/tube. The intra- and inter-assay coefficients of variations were, respectively, 7 and 9% for AVP, and 8 and 12% for OT, respectively. AVP and OT recoveries average 70% in our laboratory. All plasma samples were processed together.

### Immunohistochemistry and double labeling experiments

At the conclusion of the study, the animals were anesthetized and ventilated with a mixture of isoflurane and oxygen as described. A middle abdominal incision was made and the fetal head and neck were exposed. A 16-gauge needle was inserted into the fetal carotid artery for perfusion with 0.1 M phosphate buffered saline followed by 4% paraformaldehyde in 0.1 M phosphate buffer under anesthesia. The perfusion period was about 5–7 min, and the fetus was decapitated during perfusion. The brain was removed immediately following perfusion. Post-fixation was performed in the paraformaldehyde solution for 12 h, after which the brain was placed in 20% sucrose overnight. Twenty-micrometer coronal sections were cut through the fetal brain on a cryostat. Every other sections of the SON and PVN in the hypothalamus were used for c-fos immunoreactivity (FOS-ir) staining using the avidin-biotin-peroxidase technique. The tissue sections were incubated on a gentle shaker overnight at 4°C in the primary antibody (1:15,000, Santa Cruz Biotechnology, Santa Cruz, CA). The polyclone FOS primary antibody has been raised from rabbits against the N-terminal sequence of the FOS protein. The sections were further incubated in a goat anti-rabbit serum (1:400) for 1 h and then processed using the Vectastain ABC kit for 1 h (Vector Labs, Burlingame, CA) at room temperature. Immediately following FOS immunostaining, tissue sections were used for double immunostaining by using AVP or OT antibody. The method of double labeling was the same as reported [25]. The sections were then treated with 1 mg/ml diaminobenzidine tetrahydrochloride (Sigma-Aldrich, St. Louis, MO) (0.02% hydrogen peroxide). All sections were mounted on slides, dehydrated in alcohol, and then cover slipped.

### Data Analysis

The number of FOS-ir positive cells in the brain was evaluated in a qualitative and blinded manner as reported [51]. The number of FOS-ir positive cells in the all stained sections for the SON and PVN was counted. A repeated measures ANOVA was used to determine differences over time and effects of the treatments. Comparison before and after the treatments was determined with one-way ANOVA followed by the Tukey *post hoc *test or Student's *t*-test. All data were expressed as mean ± SEM. Statistic significance was accepted at *P *< 0.05.

## Authors' contributions

ZX conceived the study and LS, CM, FZ, YZ executed the experiments. LS, CM, FZ contributed equally to this work. All of the authors contributed either to the experimental design, or data interpretation, or the writing of the manuscript.

NOTE: The Departments of Human Sport Science, Beijing Sport University and the Perinatal Biology Center, Soochow University School of Medicine, both contributed eaqually to this article.  
